# Cacao Cultivation under Diverse Shade Tree Cover Allows High Carbon Storage and Sequestration without Yield Losses

**DOI:** 10.1371/journal.pone.0149949

**Published:** 2016-02-29

**Authors:** Yasmin Abou Rajab, Christoph Leuschner, Henry Barus, Aiyen Tjoa, Dietrich Hertel

**Affiliations:** 1 Plant Ecology and Ecosystems Research, Albrecht-von-Haller Institute for Plant Sciences, University of Göttingen, Göttingen, Germany; 2 Department of Agrotechnology, Faculty of Agricultural Sciences, Tadulako University, Palu, Sulawesi Tengah, Indonesia; North Carolina State University, UNITED STATES

## Abstract

One of the main drivers of tropical forest loss is their conversion to oil palm, soy or cacao plantations with low biodiversity and greatly reduced carbon storage. Southeast Asian cacao plantations are often established under shade tree cover, but are later converted to non-shaded monocultures to avoid resource competition. We compared three co-occurring cacao cultivation systems (3 replicate stands each) with different shade intensity (non-shaded monoculture, cacao with the legume *Gliricidia sepium* shade trees, and cacao with several shade tree species) in Sulawesi (Indonesia) with respect to above- and belowground biomass and productivity, and cacao bean yield. Total biomass C stocks (above- and belowground) increased fivefold from the monoculture to the multi-shade tree system (from 11 to 57 Mg ha^-1^), total net primary production rose twofold (from 9 to 18 Mg C ha^-1^ yr^-1^). This increase was associated with a 6fold increase in aboveground biomass, but only a 3.5fold increase in root biomass, indicating a clear shift in C allocation to aboveground tree organs with increasing shade for both cacao and shade trees. Despite a canopy cover increase from 50 to 93%, cacao bean yield remained invariant across the systems (variation: 1.1–1.2 Mg C ha^-1^ yr^-1^). The monocultures had a twice as rapid leaf turnover suggesting that shading reduces the exposure of cacao to atmospheric drought, probably resulting in greater leaf longevity. Thus, contrary to general belief, cacao bean yield does not necessarily decrease under shading which seems to reduce physical stress. If planned properly, cacao plantations under a shade tree cover allow combining high yield with benefits for carbon sequestration and storage, production system stability under stress, and higher levels of animal and plant diversity.

## Introduction

Tropical deforestation and decreasing carbon sinks are one of the major drivers increasing the concentration of atmospheric carbon dioxide (CO_2_), thereby enforcing global climate change (e.g.[1–5]). A current hotspot of rainforest conversion is Southeast Asia and in particular Indonesia [3,5–8], which lost ~158,000 km^2^ of its forest cover between 2000 and 2012 [8,9]. Indonesia’s carbon emissions reached 105 Tg C yr^-1^ between 2000 and 2005 [10] and the nation is the world’s third largest CO_2_ emitter by now [9]. Main driver of deforestation in this region is the conversion into agricultural cultivation systems (e.g. [3,7,9,11]), notably palm oil, cocoa, and rubber. Cacao (*Theobroma cacao* L.) is a crop of the humid tropical lowlands, which is mostly grown by smallholders. Due to the steadily increasing demand for chocolate [12,13], the world cocoa production has increased to ~5 million t in 2012 [FAO Statistical Database; http://faostat.fao.org] and cacao ranges currently as one of the most important perennial cash crops worldwide. In Sulawesi (Indonesia), where this study was conducted, the cultivation area of cacao expanded rapidly in the 1980s and 1990s; about 50% of the recent cacao cultivation area is located on former forested land [12]. Within the Indonesian archipelago, about 65% of Indonesia’s cacao production is generated on Sulawesi [14,15]. Since cacao is an understory rainforest species, it has traditionally been planted beneath the thinned canopy of primary or old secondary forest [12]. In Indonesia, this traditional cultivation system has increasingly been altered by removing the shade trees. In many cases, fast-growing and nitrogen-fixing shade trees like *Gliricidia* ssp. or *Erythrina* ssp., or trees which provide edible fruits, timber or other valuable goods were planted instead. Due to the shade requirement of young cacao plants, cacao is still cultivated under shade tree cover in the first years. But nowadays, shade trees are often completely removed when the cacao matures, because farmers wish to increase cacao bean yield (e.g. [12,13,16–19]). The rationale is to reduce assumed competition for light, water and nutrients between cacao and shade trees (e.g. [12,17,20]). This change in cultivation practice may have a number of negative consequences, notably losses in biodiversity, increased soil erosion due to diminished protection from heavy rain, and largely reduced carbon storage in biomass (e.g. [12,21,22]). Moreover, various monetary and non-monetary ecosystem services provided by the shade trees are no longer available to the local community, among them the supply of timber, fuel, and fruit production [19]. Even though recent research in tropical agroforests has addressed these benefits, not much is known about differences in carbon storage and carbon sequestration through net primary production (NPP) in cacao agroforests differing in shade tree cover and diversity. Even fewer studies have dealt with belowground carbon stores and C turnover of cacao agroforests and their dependence on variation in canopy cover.

The aim of the present study was to compare cacao cultivation systems from zero to high shade intensity with respect to biomass, carbon stores and productivity. In a region of Central Sulawesi with rapid expansion of cacao cultivation in recent time, where shaded and non-shaded cacao production systems co-occur in close neighborhood, we compared three widespread systems (non-shaded cacao monoculture, cacao with the legume *Gliricidia sepium* (Jacq.) as dominant shade tree, and cacao with relatively dense cover of several shade tree species) with respect to above- and belowground biomass and related carbon (C) stores, and C sequestration with above- and belowground net primary production (NPP) in each three replicate plots. We tested the hypotheses that (1) increasing shade tree abundance and diversity increases above- and belowground carbon storage and productivity, and that (2) increasing shade tree cover decreases cacao bean yield. By quantifying the biomass carbon pools and NPP of the different systems and comparing it with natural forest, we further wanted to assess the role shaded cacao cultivation systems can play in the regional carbon cycle. To our knowledge, this is the first study investigating three co-existing cacao cultivation systems (structurally simple monoculture to complex multi-species agroforest) under equal climatic and soil conditions with a focus on the carbon cycle and cacao bean yield.

## Materials and Methods

Our study complies with the current laws of Indonesia and Germany and with international rules. The research permit for the fieldwork in Indonesia was issued by RISTEK (Kementerian Riset Dan Teknologi) with the permit number: 275/SIP/FRP/SM/VII/2013. The study itself was carried out on private land. The owners of the land gave their permission to conduct the study on these sites, thus, no specific permissions were required for these locations. The field studies did not involve endangered or protected species.

### Study Site Description and Study Plot Selection

**Fig 1:**
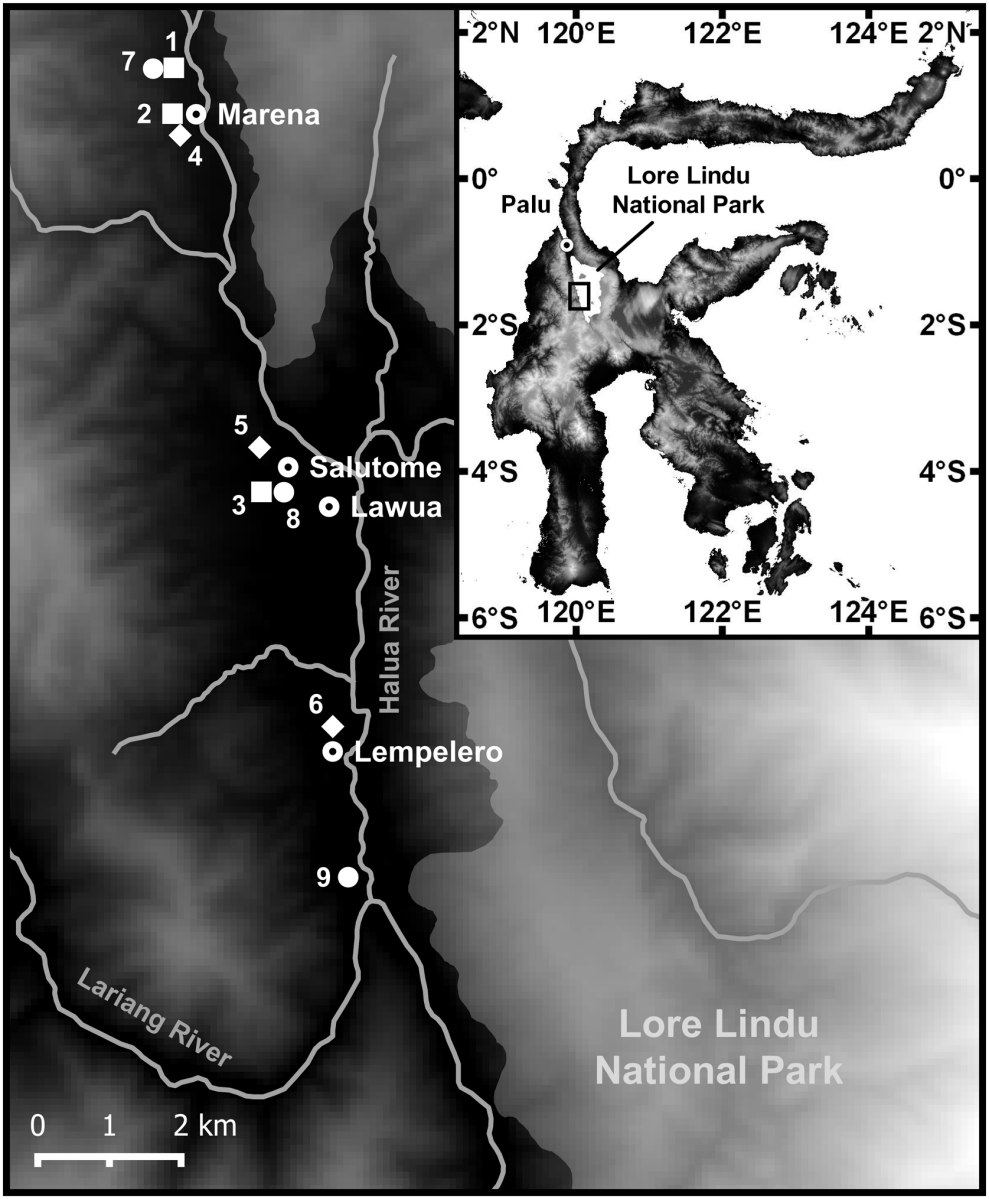
Map of the study region.

The study was conducted in the Kulawi valley in Central Sulawesi, Indonesia, in vicinity of the western border of Lore Lindu National Park (01°30´S, 120°02´E) (Fig 1). Annual mean temperature in the region was 25°C, annual mean precipitation 2165 mm yr^-1^ [23] without a distinct seasonality during the study time. We studied three different cacao cultivation systems, (i) monoculture of cacao (*Theobroma cacao* L.) (‘Cacao-mono’), (ii) cacao planted with the N-fixing legume tree *Gliricidia sepium* (Jacq.) (‘Cacao-*Gliricidia’*), and (iii) cacao cultivated with several different shade tree species (‘Cacao-multi’). Three study plots of approx. 20 m x 20 m per cultivation system type were selected on private land between the villages Marena and Lempelero in the South of Kulawi valley (Fig 1 and Table 1). Apart from the necessary agreement of the plot owners, plot selection criteria were sufficient comparability in terms of topography (only low inclination), soil texture (sandy to clayey loam) and chemistry (Cambic Umbrisols with comparable pH, base saturation and C/N ratio, Table 1). Soil chemical parameters were measured from six randomly selected soil samples taken at each study site with a soil corer (5 cm in diameter) down to a depth of 60 cm (0–10 cm, 10–20 cm, 20–40 cm, and 40–60 cm). The total carbon and nitrogen concentrations were determined in a CN auto-analyzer (Vario EL III, Hanau, Germany) the total P concentration with ICP-OES analysis after HNO_3_ digestion, the plant-available cation concentrations after NH_4_Cl extraction and subsequent element analysis in the percolate by ICP-OES. To estimate the total carbon pool in the upper 60 cm of the soil, we used data of the bulk density of the soil and the soil organic carbon content. Large variation was found for available P (resin P), which may partly be a result of different time spans since the last fertilization in the plots.

**Table 1:** Location and soil characteristics of the nine study sites grouped into the three cultivation systems investigated in the Kulawi valley (Sulawesi, Indonesia).

Cultivation system	Cacao-mono			Cacao-*Gliricidia*			Cacao-multi		
Plot No.	Plot 1	Plot 2	Plot 3	Plot 4	Plot 5	Plot 6	Plot 7	Plot 8	Plot 9
Plot location	Marena	Marena	Lawua	Marena	Salutome	Lempelero	Marena	Lawua	Lempelero
Plantation establishment	1989	1989	1997	1993	1996	1999	1989	1999	1991
**Location characteristics**									
Coordinates	1.559°S 120.022°E	1.567°S 120.024°E	1.611°S 120.034°E	1.559°S 120.022°E	1.608°S 120.033°E	1.642°S 120.042°E	0.916°S 119.877°E	1.611°S 120.036°E	1.661°S 120.044°E
Elevation (m a.s.l.)	556	567	428	571	449	387	551	397	413
Inclination (%)	ca. 1.5	7.8	4.6	ca. 8.0	6.6	9.1	ca. 1.0	1.0	1.2
**Soil properties**									
pH H_2_O (KCl)	5.9 (4.8)	5.3 (4.4)	5.5 (4.2)	5.2 (4.3)	5.4 (4.2)	5.5 (4.1)	5.7 (4.6)	6.1 (4.9)	6.1 (5.1)
Exchange capacity (μmolc g^-1^)	175.1 ± 12.1	66.7 ± 4.6	166.9 ± 14.5	78.5 ± 7.2	73.9 ± 5.9	108.4 ± 12.6	160.4 ± 15.4	185.8 ± 14.0	181.6 ± 22.0
Base saturation (%)	98.6 ± 0.5	87.6 ± 1.1	96.4 ± 1.46	81.6 ± 5.2	85.7 ± 3.6	93.7 ± 1.5	96.5 ± 2.1	98.9 ± 0.5	98.7 ± 0.5
P_resin_ (mg P kg^-1^)	24.5 ± 4.1	8.2 ± 1.8	20.7 ± 3.1	7.0 ± 0.4	21.4 ± 4.2	10.1 ± 1.0	29.2 ± 7.4	80.7 ± 7.2	67.8 ± 9.2
C_org_ (g kg^-1^)	21.9 ± 2.1	18.6 ± 1.0	18.7 ± 1.9	19.4 ± 1.1	16.1 ± 1.4	12.3 ± 2.2	23.1 ± 3.2	23.3 ± 1.4	31.5 ± 1.9
N_total_ (g kg^-1^)	2.1 ± 0.2	2.0 ± 0.1	1.9 ± 0.1	1.9 ± 0.1	1.7 ± 0.1	1.3 ± 0.2	2.2 ± 0.2	2.2 ± 0.1	2.6 ± 0.1
C_org_/N_org_ (g g^-1^)	10.5 ± 0.4	9.3 ± 0.1	9.7 ± 0.3	10.0 ± 0.3	9.2 ± 0.2	9.4 ± 0.3	10.4 ± 0.4	10.8 ± 0.5	11.9 ± 0.3
C_pool_ (Mg ha^-1^)	15.1 ± 1.8	30.1 ± 3.1	19.4 ± 1.1	28.8 ± 1.1	29.7 ± 1.8	21.2 ± 2.4	19.7 ± 2.2	29.9 ± 2.5	38.2 ± 1.6
Soil type	Cambic Umbrisol	Cambic Umbrisol	Cambic Umbrisol	Cambic Umbrisol	Cambic Umbrisol	Cambic Umbrisol	Cambic Umbrisol	Cambic Umbrisol	Cambic Umbrisol
Soil texture	sandy-silty loam	silty loam	silt	silty loam	sandy loam	sandy loam	silty sand to clay loam	silt	sandy-silty loam

Cacao-mono: cacao in monoculture, Cacao-*Gliricidia*: cacao growing under *Gliricidia sepium*, Cacao-multi: cacao agroforests with multi-species shade layer. The soil parameters are given as means ± SE and refer to the topsoil (0–10 cm soil depth) except for the soil carbon stock C_pool_, which refers to the whole sample profile (0–60 cm soil depth).

### Aboveground Stand Structure

A stand inventory was conducted in all nine plots at the beginning of the study in summer 2011. Tree height was determined using an ultrasonic Vertex III height meter (Haglöf, Langsele, Sweden) and stem diameter at breast height (dbh, at 130 cm) was measured using a measuring tape. In cases of some cacao and *Gliricidia* trees, where the stem branched before 130 cm height, the stem diameter was measured at the next possible height. Subsequently, the diameter was extrapolated to 1.3 m using species-specific linear allometric regressions between height and diameter, which were obtained by measuring the stem diameter of 10 trees per species in height-intervals of 10 cm.

The diversity of woody species in the plots was quantified with Shannon-Wiener’s H’ (eq 1):

$$\text{H}=-\sum {\text{(p}}_{\text{i}}\times {\text{log p}}_{\text{i}}),$$ (1)

with H being the diversity index, p_i_ representing the relative abundance of species i (p_i_ = n_i_/N), n_i_ the number of individuals per species i, and N the total number of species per study plot.

### Aboveground Biomass (AGB) and Belowground Biomass (BGB)

The AGB of cacao trees was calculated using the allometric equation of Beer et al. [24], see eq 2). For the AGB estimation of *Gliricidia sepium* and all other shade tree species, we used the allometric equation of Chave et al. [25] for tropical moist forest trees (eq 3). The AGB of one single coconut palm individual (*Cocos nucifera* L.) in the plots was calculated after Hairiah et al. [26] using eq 4. For banana (*Musa* sp.), we used eq 5 after Van Noordwijk and Mulia [27]:

AGB = -0.0376 + (0.133 BA) (2)

$$\text{AGB }=\text{ exp }(\text{2.557 }+\text{ 0.940 ln }(\rho {\text{D}}^{2}\text{H}))\;$$ (3)

$$\text{AGB }=\;\pi {\text{D}}^{2}\text{H}\rho /40$$ (4)

$$\text{AGB }=\text{ 0}{\text{.03 D }}^{\text{2.13 }}$$ (5)

with AGB being the estimated aboveground biomass in kg per tree (including stem, branch wood and leaf biomass), BA stem basal area at breast height (in cm²), D stem diameter at breast height (in cm), H total tree height (in m), and ρ wood density (in g cm^-3^). Wood density values for most of the species were obtained from Kotowska et al. [28], who measured ρ in the same study plots. In cases of tree species, for which wood density data was not available from this source, we used data from the World Agroforestry Centre [http://db.worldagroforestry.org/wd, January 2014] and from other literature sources [29,30]. In two cases, where no information on wood density from these sources was available, we used the plot average of wood density as an estimate (e.g. [15,30,31].

The BGB was estimated indirectly from the AGB using eq 6 after Cairns et al. [32].

BGB = exp (-1.0587 + 0.8836 ln (AGB)) (6)

As this equation delivers data on coarse root and root stock biomass, but not on fine root biomass, we added the standing fine root biomass from our own inventory down to 3 m soil depth to the BGB on each plot (see description below).

To obtain profile totals of standing fine root biomass, root inventory data from the upper soil (0–60 cm) and from deep soil pits (0–300 cm) were obtained and combined as follows; For recording the standing fine root mass, twelve randomly selected soil samples were taken at each study site with a soil corer (3.5 cm in diameter) down to a depth of 60 cm (0–10 cm, 10–20 cm, 20–40 cm, and 40–60 cm).

In the laboratory, the root samples were soaked in water and cleaned of soil residues using a sieve with a mesh size of 1 mm. Large root fractions (>10 mm in length) were picked out by hand. Living and dead rootlets were distinguished under the stereomicroscope by color, root elasticity, and the degree of cohesion of cortex, periderm, and stele following the method of Leuschner et al. [33]. For half of the samples, an additional, more detailed analysis of small fine root particles (<10 mm in length) was conducted applying a method introduced by Van Praag et al. [34] and modified by Hertel and Leuschner [35]. The mass of small rootlets was extrapolated to the entire sample by a regression analysis of small rootlets to large rootlets. Alternatively, a mean ratio of small to large root fractions was used if not enough data for performing a regression analysis was available.

In order to analyze the standing fine root biomass also in the subsoil to 300 cm depth, soil pits were excavated at each study site. In the cacao monoculture systems, each two soil pits ca. 80 cm distant to randomly selected cacao trees were excavated. In case of the shaded cacao agroforests, two soil pits each for two cacao trees and two shade trees were excavated (i.e. average half way between the stems). Root biomass was recorded by extracting soil monoliths of 30 cm x 30 cm x 20 cm size (l x w x d) down to 120 cm in the soil profile, and of 30 cm x 30 cm x 40 cm in the 120–300 cm layer, respectively. The roots were separated by species and into fine roots (roots <2 mm in diameter), large roots (2–5 mm in diameter), and coarse roots (>5 mm in diameter).

In plot #6, the deep soil pits could only be excavated to a depth of 100 cm due to standing water in the pits after heavy rainfall. In this case, we used the mean fine root biomass values for 100–300 cm depth from the other two plots of this cultivation system (plots # 4 + 5) to calculate the profile total of fine root biomass.

Roots of grasses and herbs were easily distinguishable from tree fine roots by their smaller diameter, lighter color and the absence of a lignified periderm, but these roots were ignored as the proportion of herb and grass root mass was below 5% in all plots. In some of the study plots, roots of trees growing outside the plots or belonging to dead tree stumps of the cut shade trees were found. In most cases, this fraction did not exceed 5%, except for plot #1 (40%), plot #2 (11%) and plot #5 (10%). In the analysis of standing fine root biomass, these root fractions were included.

### Above- and Belowground Net Primary Production (NPP)

Annual above- and belowground NPP was quantified based on the measurement of annual cacao bean yield and total cacao fruit production, annual aboveground woody growth, litter production, and fine and coarse root and root stock biomass production.

For analyzing cacao bean yield and total cacao fruit production (beans and pods), we used data from each 20 cacao trees in the cacao monocultures and from each 10 cacao trees in the shaded agroforest systems that were harvested in a manner as done by the local farmers. In this way, ripe cacao pods were cut every two weeks over a 12-month period and the fresh weight measured directly in the field. For obtaining dry bean weight and dry pod weight, 10 representative pods per study plot were selected in all size classes to determine the ratio of whole fruit fresh weight to dry bean weight and dry pod weight in every plot. To do so, fresh pods were weighed, all fruit components (skin, seeds and fruit pulp) dried separately (70°C, 72h) and their dry weight determined in the laboratory of the Tadulako University in Palu.

Aboveground woody biomass production was calculated from stem increment data obtained by repeated reading of manual dendrometer tapes (UMS, Munich, Germany) that were installed at breast height during a 12-month period on each 20 tree individuals per study plot. In the shaded cacao systems, each 10 cacao and 10 shade trees were mounted. To calculate the mean annual wood production of the 20 tree individuals of a plot, the diameter increment was applied to the allometric biomass equations given above. The calculated annual wood production rates per tree were extrapolated to all other tree individuals of the respective species or family in a plot. For two tree individuals of species not included in the dendrometer study, we applied plot means of annual basal area increment rate. The few coconut and banana trees were ignored because they do not show secondary stem diameter growth and we lack data on aboveground biomass production.

In order to measure annual leaf and fine litter production, 10 litter traps (size approximately 75 cm x 75 cm) per study site were installed. The litter was collected monthly and sorted at species level into leaves and other fine litter fractions (i.e. flowers, fruits and small twigs). The litter fractions were dried for 72 h at 70°C and weighed in the laboratory in Palu. The litter of trees not growing inside the plot area was added to cacao trees in case of monocultures or to the shade trees in case of shaded cacao cultivation systems, assuming that approximately the same amount of litter should move into the plot by wind as is carried out in the considered time interval. Assuming that monthly leaf and fine litter fall equals monthly leaf and fine litter production, we took the annual litter mass in the plots as litter production [15]. Here, only the data for leaves of cacao and shade trees and the remaining litter components not sorted by species are shown, because leaves of cacao and shade trees made up the largest part of the litter mass in the study sites. Traditionally, all cacao trees are pruned regularly. During the study period, farmers left out pruning to avoid differences between the study sites, except of one single pruning event at the end of the study phase, where all farmers pruned at the same time. Unfortunately, the mass of cut twigs and leaves at this event could not be recorded, but this should not have had notable effects on our data.

Fine root production was estimated in the different cultivation systems by conducting an ingrowth core experiment with local soil material according to the methodology described by Persson [36] and Hertel and Leuschner [35]. At 10 randomly chosen locations in each of the nine stands, soil cores were taken (3.5 cm in diameter) from the first 30 cm of the soil. All macroscopically visible live and dead root material was extracted by hand in the field. The remaining soil material was replaced into the hole and the location marked with plastic tubes. Care was taken that the structure and density of the soil in the cores was conserved as much as possible. The samples were recollected with the same soil corer after 10 months, and the extracted core sliced into the soil layers 0–10 and 10–30 cm depth. In the laboratory of the Tadulako University of Palu, root biomass was extracted as described above. Following Vogt et al. [37] and Hertel and Leuschner [35], we calculated fine root production in the cores as the increase in root biomass from the start of root recolonisation (in our study 1 month after installation) until harvest. Fine root growth in the cores during the recolonisation period was extrapolated to 1 year and expressed in g m^-2^ yr^-1^.

The production of coarse root and root stock biomass was calculated from the increase in aboveground woody biomass from the beginning to the end of the study using the allometric equation after Cairns et al. [32] (see above). The difference was taken as annual coarse root and root stock biomass production.

### Carbon Pools in Biomass and Production

All above- and belowground biomass and production values were converted into carbon stored in plant biomass. The calculation was done based on the C concentration detected in the different plant fractions. Samples of stem wood, fine roots (diameter <2 mm), coarse roots (diameter >2 mm) and the different litter fractions were analyzed in a CN auto-analyzer (Vario EL III, Hanau, Germany) at the University of Göttingen, Germany. Only for the C stock present in the cacao bean yield, we used carbon content data from literature [38]. The carbon pool of the whole cacao pods was calculated by summing up the carbon stock of the cacao bean seeds from literature and the carbon stock available in the cacao pods without seeds taken from the litter traps. Cacao pods that fell into the litter traps were still too small to contain seeds.

### Statistical Analyses

All data were tested for Gaussian distribution using a Shapiro–Wilk test. The majority of the datasets showed a non-Gaussian distribution and could not satisfyingly be transformed. Thus, differences between the cultivation systems were analyzed for all parameters using non-parametric analyses of variance (Kruskal–Wallis test) and a subsequent Mann–Whitney two-sample test (Wilcoxon U-test). These calculations as well as Pearson correlation analyses were done with the software package SAS 9.3 (version 9.3, SAS Institute, Cary, NC, USA). Regression analyses were conducted with the software package SigmaPlot (version 11.0, Systat Software Inc.). For analyzing interrelations between tree species diversity, stand structure, carbon sequestration and cacao bean yield, we conducted a Principle Components Analysis (PCA) with the package CANOCO, version 4.5 (Biometris, Wageningen, The Netherlands).

## Results

### Aboveground Stand Structure

The Shannon diversity index H’ increased from 0 to 0.4 in the sequence cacao monocultures—Cacao-*Gliricidia* systems—Cacao-multi shade-tree systems (Table 2). In parallel, canopy cover increased from 50 to 93%, total tree density from 900 to 1700 ha^-1^, and stand basal area from 12.6 to 34.6 m^2^ ha^-1^. The *Gliricidia* shade trees were 3–4 meters taller than the cacao trees; in the multi shade-tree systems, several shade tree species were even taller than the *Gliricidia* trees and had much larger stem diameters. Total stand basal area was nearly three times larger in the multi shade-tree systems than in the cacao monocultures, while the total number of shade trees was smaller than in the *Gliricidia* systems (Table 2). Stem density of cacao was 30–40% higher in the two shaded systems than in the cacao monocultures (2370 and 2540 vs. 1800 ha^-1^, respectively).

**Table 2:** Aboveground stand structural properties of the three cultivation systems in the Kulawi valley (Sulawesi, Indonesia) (means ± SE of each three stands).

Cultivation system	Tree identity	Canopy cover (%)	Tree density (no. ha^-1^)	Stem density (no. ha^-1^)	Stand basal area (m^2^ ha^-1^)	Stem diameter (cm)	Tree height (m)	Shannon-Index H’
Cacao-mono	Cacao	-	892 ± 128 a	1804 ± 264 a	12.6 ± 2.3 ab	8.6 ± 0.5 a	5.1 ± 0.01 a	-
	Shade trees	-	-	-	-	-	-	-
	All	50.0 ± 15.3 A	892 ± 128 A	1804 ± 264 A	12.6 ± 2.3 A	8.6 ± 0.5 A	5.1 ± 0.01 A	0 ± 0 A
Cacao-*Gliricidia*	Cacao	-	1047 ± 150 a α	2538 ± 489 a α	9.2 ± 0.9 a α	6.5 ± 0.5 b α	4.6 ± 0.2 b α	-
	Shade trees	-	428 ± 140 a β	718 ± 41 a β	4.4 ± 0.6 a β	7.4 ± 0.4 a α	8.5 ± 1.2 a β	-
	All	60.0 ± 11.5 A	1497 ± 194 B	3277 ± 443 B	13.8 ± 1.1 A	6.9 ± 0.4 B	5.6 ± 0.2 B	0.3 ± 0.1 B
Cacao-multi	Cacao	-	1384 ± 288 a α	2368 ± 433 a α	14.0 ± 1.4 b α	7.9 ± 0.4 ab α	5.1 ± 0.1 ab α	-
	Shade trees	-	357 ± 118 a β	541 ± 290 a β	20.6 ± 1.6 b β	24.6 ± 3.5 b β	11.9 ± 1.8 a β	-
	All	93.3 ± 1.7 B	1741 ± 343 B	2909 ± 668 AB	34.6 ± 2.2 B	10.0 ± 0.6 A	6.3 ± 0.2 C	0.4 ± 0.05 B

Different capital letters indicate statistically significant differences between the agroforestry systems (‘all’), lower case Latin letters significant differences of the different agroforest components (cacao or shade trees, or both) between the cultivation systems, and lower case Greek letters significant differences between cacao and shade trees within a cultivation system (P < 0.05).

### Aboveground and Belowground Biomass and Related C Pools

**Fig 2:**
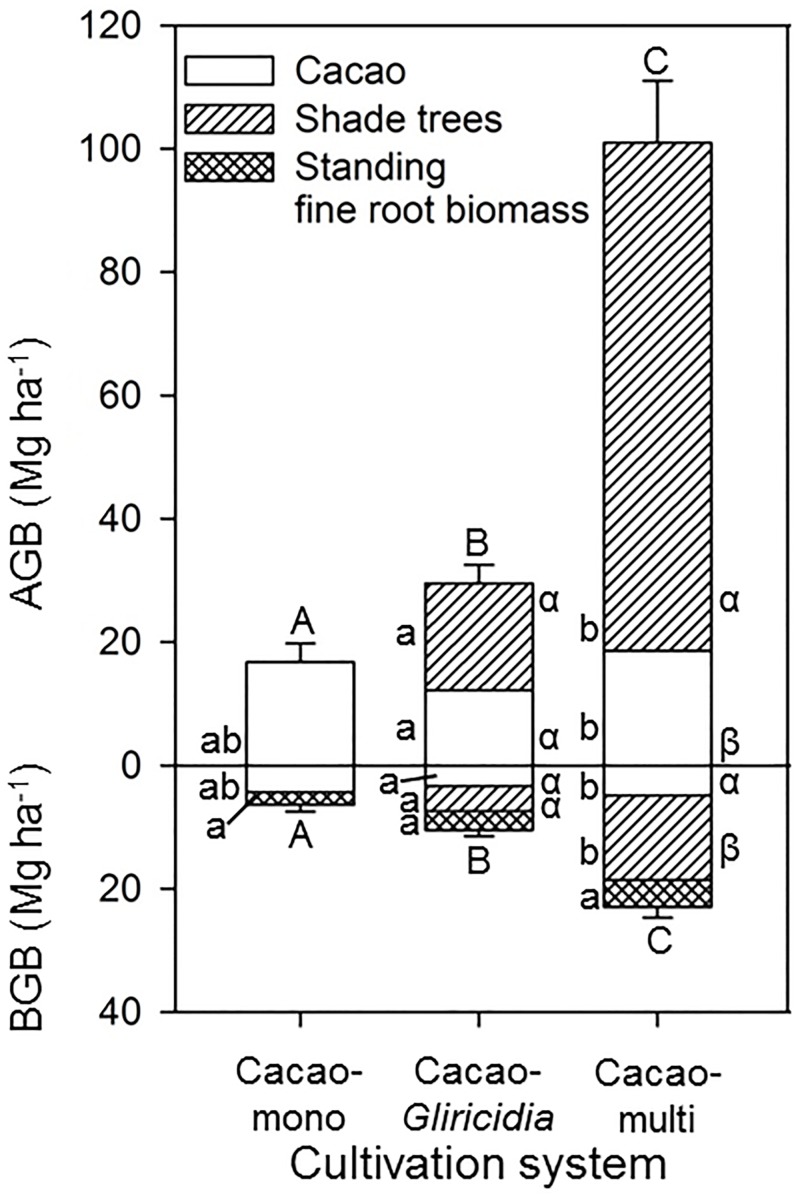
Above- and belowground biomass, including standing fine root biomass of the three different cacao cultivation systems. Different capital letters indicate statistically significant differences between the agroforestry systems, lower case Latin letters significant differences of the different tree groups between the cultivation systems and lower case Greek letter significant differences between cacao and shade trees within a cultivation system (*P* < 0.05).

Total aboveground biomass increased more than fivefold from ~17 Mg ha^-1^ in the monocultures to 30 Mg ha^-1^ in the Cacao-*Gliricidia* plantations and to 100 Mg ha^-1^ in Cacao-multi systems (Fig 2). Total belowground biomass including the standing fine root biomass in the 0–300 cm soil profile showed a similar increase from 6.4 Mg ha^-1^ in the monocultures to 10.5 Mg ha^-1^ in Cacao-*Gliricidia* systems and to 22.9 Mg ha^-1^ in the multi shaded tree systems. The biomass and carbon contributed by the cacao trees was somewhat lower in the Cacao-*Gliricidia* stands than in the other two cultivation systems (Fig 2 and Table 3). Total biomass carbon was nearly six times larger in the multi shade-tree systems than in the monocultures.

**Table 3:** Above- and belowground carbon stocks and the shoot:root carbon ratio (means ± SE).

Cultivation system	Tree identity	Aboveground carbon stock	Belowground carbon stock	Total carbon stock	Shoot:root ratio
		(Mg ha^-1^)	(Mg ha^-1^)	(Mg ha^-1^)	
Cacao-mono	Cacao	7.7 ± 1.4 ab	1.9 ± 0.4 a	9.7 ± 1.8 ab	4.1 ± 0.1 a
	Shade trees	n.a.	n.a.	n.a.	n.a.
	All	7.7 ± 1.4 A	2.8 ± 0.5 A	10.6 ± 1.9 A	2.8 ± 0.3 A
Cacao-*Gliricidia*	Cacao	5.6 ± 0.6 a α	1.5 ± 0.1 a α	7.1 ± 0.7 a α	3.7 ± 0.1 b α
	Shade trees	8.4 ± 0.9 a β	1.6 ± 0.1 a α	10.5 ± 0.8 a β	4.2 ± 0.8 a α
	All	14.0 ± 1.4 B	4.4 ± 0.4 B	18.4 ± 1.8 B	3.2 ± 0.1 A
Cacao-multi	Cacao	8.6 ± 0.8 b α	2.1 ± 0.2 a α	10.2 ± 0.7 b α	5.3 ± 0.8 ab α
	Shade trees	39.0 ± 5.4 b β	5.9 ± 0.5 b β	44.9 ± 5.9 b β	6.6 ± 0.4 b α
	All	47.6 ± 4.8 C	9.8 ± 0.7 C	57.4 ± 5.5 C	4.9 ± 0.1 B

Only for the tree group ‘all’, fine root data is included. Given are means and standard errors. Different capital letters indicate statistically significant differences between the agroforestry systems, lower case Latin letters indicate statistically significant differences of the different tree groups between the cultivation systems and lower Greek case letter indicate statistically significant differences between cacao and shade trees within a cultivation system (*P* < 0.05).

Standing fine root biomass (0–300 cm profile) increased, although not significantly, with increasing shade tree cover in the three systems (206, 301 and 432 g m^-2^, respectively) with more than two times larger totals in the Cacao-multi plots than in the monocultures (data not shown). The biomass increase was greater in the aboveground than the belowground compartment, leading to a shoot: root ratio increase from 2.8 to 4.9 from the monocultures to the diverse multi shade-tree systems (Table 3).

### Net Primary Production and Its Components

**Fig 3:**
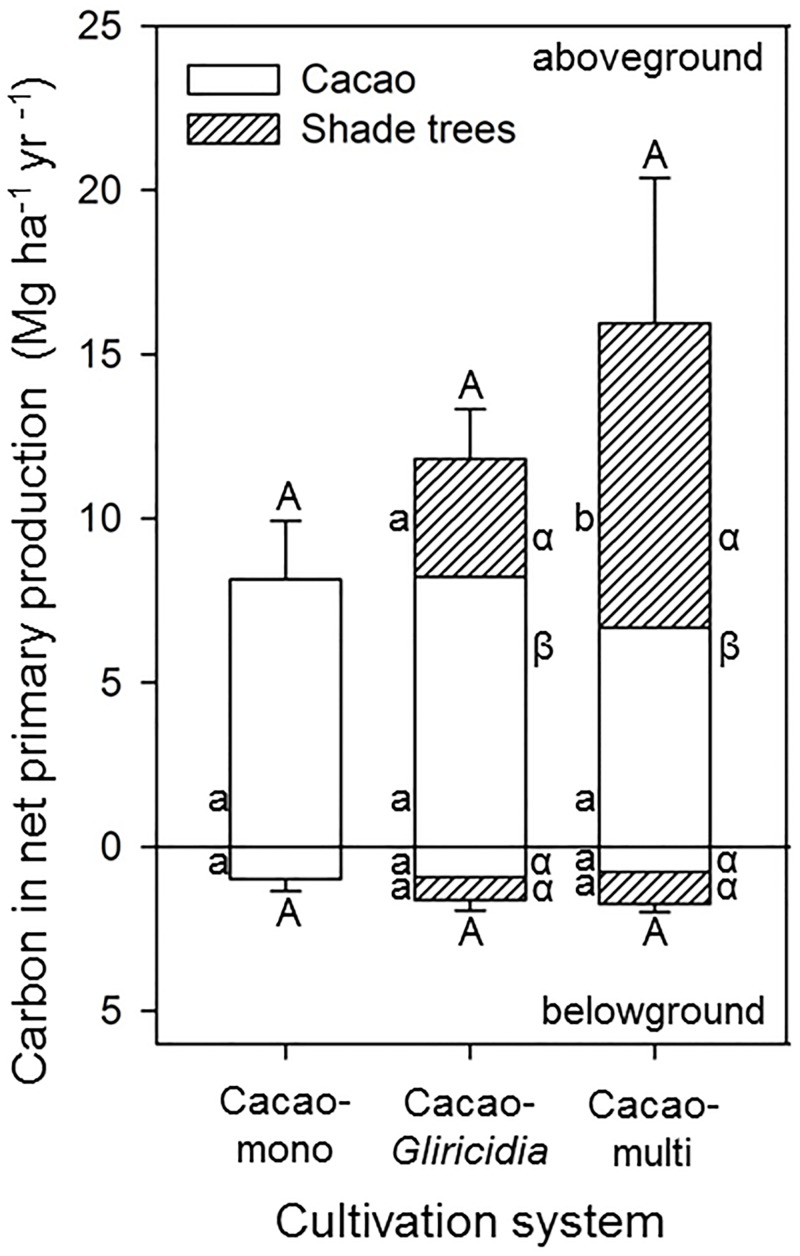
Above- and belowground carbon production of the three different cacao cultivation systems. Different capital letters indicate statistically significant differences between the agroforestry systems, lower case Latin letters significant differences of the different tree groups between the cultivation systems and lower case Greek letter significant differences between cacao and shade trees within a cultivation system (P < 0.05).

Total (above- and belowground) net primary production nearly doubled from the monocultures to the multi shade-tree systems (19.5, 28.2 and 37.7 Mg ha^-1^ yr^-1^ in the three systems, equaling 9.1, 13.4 and 17.7 Mg C ha^-1^ yr^-1^; Fig 3 and Table 4). The increase was mainly driven by the much larger wood and coarse root production of the shaded systems than of the cacao monocultures, while the increase in litter production from the monocultures to the multi shade-tree systems was only moderate (5.3 to 9.7 Mg ha^-1^ yr^-1^); fine root production remained unchanged (1.7, 1.5 and 1.9 Mg ha^-1^ yr^-1^). The total biomass production of cacao showed a slight but non-significant decrease from the monocultures (19.5 Mg ha^-1^ yr^-1^) to the multi shade-tree systems (15.7 Mg ha^-1^ yr^-1^). Similarly, cacao fruit production (beans and pods) tended to be somewhat lower in the latter systems (8.3 vs. 9.7 Mg ha^-1^ yr^-1^) while bean production was remarkably invariant across the three cultivation systems (2.0–2.1 Mg ha^-1^ yr^-1^; Table 4). The litter production of cacao was much higher in the monocultures (5.3 Mg ha^-1^ yr^-1^) than in the two shaded systems (2.9 Mg ha^-1^ yr^-1^). When calculated per cacao tree individual, been yield decreased from 2.4 to 2.0 and 1.6 Mg ha^-1^ yr^-1^ from the monocultures to the *Gliricidia* shade system and to the multi shade-tree system (differences not significant), but this tendency was compensated by the higher cacao stem density on the plot level in the latter.

**Table 4:** Components of annual net primary production (NPP) and the associated carbon pools (in Mg ha^-1^ yr^-1^ or Mg C ha^-1^ yr^-1^) in the three cacao cultivation systems (means ± SE).

**NPP** (Mg dry matter ha^-1^ yr^-1^)										
**Cultivation system**	**Tree identity**	**Cacao bean yield**	**Cacao fruit production**	**Aboveground woody biomass production**	**Litter production**	**Fine root production** (0-60cm)	**Coarse root Biomass production**	**Total production**	**Ratio aboveground production/ belowground production**	**Ratio leaf litter production/fine root production**
Cacao-mono	Cacao	2.1 ± 0.3 a	9.7 ± 2.4 a	2.3 ± 0.8 a	5.3 ± 0.6 a	1.7 ± 0.9 a	0.5 ± 0.2 a	19.5 ± 3.9 a	9.6 ± 2.5 a	4.8 ± 1.9 a
	Shade trees			n.a.	n.a.	n.a.	n.a.	n.a.	n.a.	n.a.
	All			2.3 ± 0.8 A	5.3 ± 0.6 A	1.7 ± 0.9 A	0.5 ± 0.2 A	19.5 ± 3.9 A	9.6 ± 2.5 A	4.8 ± 1.9 AB
Cacao-*Gliricidia*	Cacao	2.1 ± 0.6 a	10.9 ± 2.8 a	3.1 ± 0.5 a α	2.9 ± 0.5 b α	1.3 ± 0.1 a α	0.8 ± 0.1 a α	19.0 ± 3.1 a α	8.3 ± 2.0 a α	2.0 ± 0.4 a α
	Shade trees			5.7 ± 1.2 a α	1.7 ± 0.4 a α	0.6 ± 0.3 a β	1.2 ± 0.2 a α	9.2 ± 1.8 a β	4.6 ± 0.6 a α	4.7 ± 2.1 a α
	All			8.8 ± 1.6 B	4.7 ± 0.9 A	1.9 ± 0.3 A	1.9 ± 0.3 B	28.2 ± 4.4 A	6.5 ± 1.2 A	2.3 ± 0.5 A
Cacao-multi	Cacao	2.0 ± 0.7 a	8.3 ± 2.2 a	2.8 ± 1.0 a α	2.9 ± 0.2 b α	1.1 ± 0.3 a α	0.6 ± 0.2 a α	15.7 ± 1.4 a α	7.8 ± 0.6 a α	2.4 ± 0.6 a α
	Shade trees			12.8 ± 1.3 b β	6.9 ± 0.8 b β	0.4 ± 0.1 a β	1.9 ± 0.3 a β	22.0 ± 1.8 b β	8.6 ± 0.7 b β	13.8 ± 1.8 b β
	All			15.6 ± 2.3 B	9.7 ± 0.6 B	1.5 ± 0.2 A	2.6 ± 0.5 B	37.7 ± 1.2 B	8.3 ± 0.6 A	5.2 ± 0.9 B
**C in NPP** (Mg C ha^-1^ yr^-1^)										
**Cultivation system**	**Tree identity**	**Cacao bean yield**	**Cacao fruit production**	**Aboveground woody biomass production**	**Litter production**	**Fine root production** (0-60cm)	**Coarse root Biomass production**	**C in total production**		
Cacao-mono	Cacao	1.2 ± 3.6 a	4.6 ± 1.2 a	1.1 ± 0.4 a	2.4 ± 0.3 a	0.7 ± 0.4 a	0.2 ± 0.1 a	9.1 ± 2.0 a		
	Shade trees			n.a.	n.a.	n.a.	n.a.	n.a.		
	All			1.1 ± 0.4 A	2.4 ± 0.3 A	0.7 ± 0.4 A	0.2 ± 0.1 A	9.1 ± 2.0 A		
Cacao-*Gliricidia*	Cacao	1.2 ± 0.6 a	5.4 ± 1.3 a	1.4 ± 0.2 a α	1.3 ± 0.2 b α	0.6 ± 0.0 a α	0.3 ± 0.1 a α	9.1 ± 1.4 a α		
	Shade trees			2.8 ± 0.6 a α	0.8 ± 0.2 a α	0.2 ± 0.1 a β	0.5 ± 0.1 a α	4.3 ± 0.8 a β		
	All			4.2 ± 0.8 B	2.2 ± 0.4 A	0.8 ± 0.3 A	0.8 ± 0.1 B	13.4 ± 2.0 A		
Cacao-multi	Cacao	1.1 ± 6.4 a	4.1 ± 1.1 a	1.3 ± 0.5 a α	1.3 ± 0.1 b α	0.5 ± 0.1 a α	0.3 ± 0.1 a α	7.4 ± 0.7 a α		
	Shade trees			6.0 ± 0.6 b β	3.2 ± 0.3 b β	0.2 ± 0.0 a β	0.8 ± 0.1 a β	10.3 ± 0.8 b β		
	All			7.3 ± 1.2 B	4.5 ± 1.1 B	0.6 ± 0.2 A	1.1 ± 0.2 B	17.7 ± 0.6 B		

Note that coarse root biomass production includes production of root stocks as well. Different capital letters indicate statistically significant differences of all tree groups in the whole soil profile between the agroforestry systems, lower case Latin letters significant differences of the different tree groups between the cultivation systems and lower case Greek letter significant differences between cacao and shade trees within a cultivation system (*P* < 0.05).

**Fig 4:**
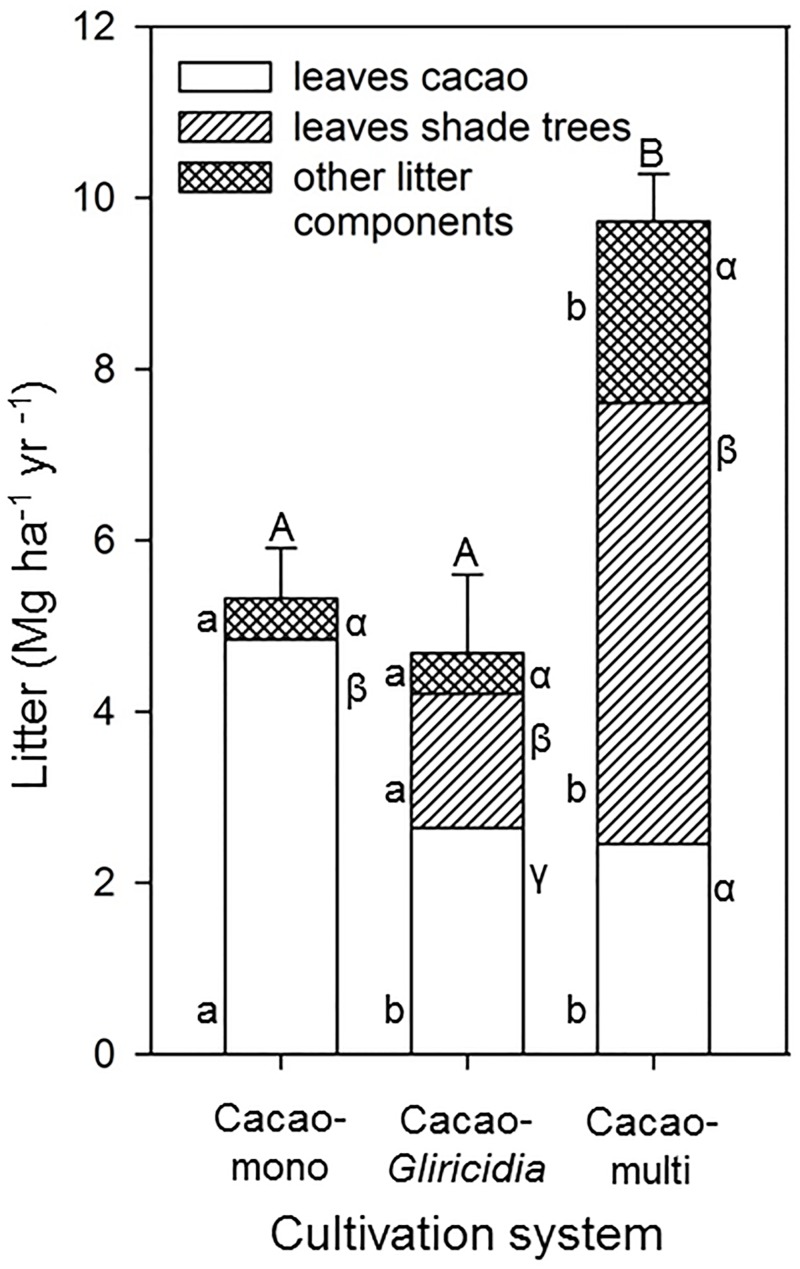
Litter production of the different cacao agroforests. Shown are the leaf litter of cacao and shade trees and the remaining litter components per cultivation system. Different capital letters indicate statistically significant differences between the agroforestry systems, lower case Latin letters significant differences of the different tree groups between the cultivation systems and lower case Greek letter significant differences between cacao and shade trees within a cultivation system (*P* < 0.05).

In the Cacao-mono and the Cacao-*Gliricidia* systems, leaf litter made up 91 and 90% of the total aboveground litter production, respectively, while in the Cacao-multi shade-tree systems, 21% of aboveground litter referred to other components (flowers, fruits, twigs, Fig 4). While total fine root production did not differ between the three cultivation systems, the fine root productivity of cacao tended to decrease from monocultural to multi shade-tree systems despite increasing cacao stem density (Table 4; difference not significant). Unexpected is that the fine root production of cacao trees was larger than that of shade trees in both shaded systems despite higher aboveground productivity of the latter, although this effect was not significant.

The ratio of above- to belowground NPP was highest in the cacao monocultures, followed by the Cacao-multi systems and lowest in the Cacao-*Gliricidia* stands (Table 4). However, while the above- to belowground NPP ratio tended to decrease for cacao trees with increasing shade tree diversity, this ratio increased in the same direction for the shade trees (only partly significant at p<0.05).

### Interrelations between Shade Tree Diversity, Stand Structure, Productivity, and Cacao Bean Yield

A Principal Components Analysis on the inter-relationships between cacao bean yield, C storage in biomass, aboveground, belowground and total NPP, as well as aboveground stand structural properties and tree species diversity (Shannon index) in the nine stands revealed a close association of the tested biomass and productivity parameters with tree density, basal area, canopy cover and also canopy layer diversity (H’), but not with cacao bean yield (Table 5). Cacao bean yield showed a close (negative) association with axis 2 (eigenvalue 0.184) but was relatively independent from the other tested biomass and productivity parameters.

**Table 5:** Results of a PCA analysis based on the plots of the three studied cacao cultivation systems and their corresponding data on cacao bean yield, carbon stores, stand structural data and diversity.

Variables	Axis 1	Axis 2	Axis 3	Axis 4
	(EV 0.6224)	(EV 0.1495)	(EV 0.0913)	(EV 0.0612)
Cacao bean yield	-0.100 (0.01)	**-0.911 (0.87)**	0.174 (0.05)	0.121 (0.05)
C in aboveground NPP	**-0.907 (0.82)**	-0.430 (0.11)	-0.152 (0.02)	0.003 (0.00)
C in belowground NPP	**-0.690 (0.48)**	-0.435 (0.00)	-0.228 (0.38)	-0.056 (0.00)
C in total NPP	**-0.915 (0.84)**	-0.178 (0.09)	0.031 (0.04)	-0.336 (0.00)
C in total biomass	**-0.894 (0.80)**	0.148 (0.00)	0.445 (0.06)	-0.094 (0.13)
Tree density	**-0.758 (0.57)**	0.195 (0.07)	-0.535 (0.00)	0.168 (0.31)
Stand basal area	**-0.907 (0.82)**	0.177 (0.00)	0.273 (0.06)	0.139 (0.02)
Canopy cover	**-0.787 (0.62)**	0.290 (0.05)	0.102 (0.20)	0.458 (0.03)
Shannon-Index	**-0.801 (0.64)**	0.492 (0.15)	-0.081 (0.01)	-0.302 (0.00)

Given are the eigenvalues (EV) of the four main axes and the loading of nine parameters on these. The values in brackets give the fraction of variance explained by the variable. The most important factors on each axis are printed bold.

Bivariate Pearson correlation analyses showed that all biomass and productivity parameters were significantly related to tree diversity in the canopy of the cacao cultivation systems except for bean yield, litter production and fine root production; the latter varied independently from the number of shade tree species present (Table 6).

**Table 6:** Pearson correlation coefficients of linear regressions between species diversity (Shannon Index) and yield and above- and belowground carbon stocks from biomass and net primary production (NPP).

Parameter	Source	Correlation coefficient	P
Shannon-Index	Cacao bean yield	-0.26	0.51
	Aboveground woody biomass production	0.77	**<0.05**
	Litter production	0.57	0.11
	Fine root production (0-60cm)	-0.16	0.68
	Coarse root biomass production	0.72	**<0.05**
	Total NPP	0.66	**<0.05**
Shannon-Index	Aboveground biomass	0.70	**<0.05**
	Belowground biomass	0.73	**<0.05**
	Total biomass	0.70	**<0.05**

## Discussion

### Shade Tree Effects on Biomass, Carbon Stores and Productivity

Total above- and belowground carbon in biomass was five times larger in the multi-shade tree systems than in cacao monoculture. With 11, 18 and 57 Mg C ha^-1^ in monoculture, Cacao-*Gliricidia* and multi-shade tree system, respectively, the measured carbon stocks were well within the range of values reported for cacao agroforests in other tropical regions (e.g. [22,39,40]). Shade trees contributed 57 and 78 percent of the total biomass carbon (above- and belowground) in the *Gliricidia* and the multi-species cultivation system, respectively. Clearly, the 57 Mg C ha^-1^ are only about one third of the biomass C, which is stored in a natural tropical forest in the region (~150 Mg C ha^-1^, [15]). Considering soil carbon stocks as well, these losses are even higher as Kessler et al. [41] reported a total above- and belowground carbon stock of 284 Mg C ha^-1^ in natural forest plots in Sulawesi. Although the soil organic carbon content from 0–60 cm increased from 22 Mg ha^-1^ to 27 Mg ha^-1^ and 29 Mg ha^-1^ in the Cacao-mono, Cacao-*Gliricidia* and Cacao-multi systems, respectively (data not shown), we could not find significant differences. Even though carbon fixation of the whole system is much lower in diverse cacao agroforests than in primary forests, nevertheless it is greater than in perennial monocultures or annual crops (e.g. [12,42,43]). The huge contribution of shade trees to biomass, carbon storage and annual carbon sequestration both above- and belowground stresses the importance of the role of shade trees in agroforestry ecosystems in our study.

The increasing canopy cover from 50% to 93% from the monocultural to the multi-species system was associated with a 6fold increase in aboveground biomass but only a 3.5fold increase in root biomass resulting in nearly a doubling of the shoot:root biomass ratio (2.8 to 4.9). A significant positive interrelation between tree species richness and both, carbon stocks in above- and belowground biomass, and in annual carbon sequestration via NPP could be shown. Nevertheless, fine root biomass in the 300 cm deep profile was more than doubled in this sequence of increasing shade tree diversity (206 to 432 g m^-2^), which must have increased the intensity of root competition. However, the differences in the increase of total aboveground biomass also suggest that the planting of tall-growing shade trees is leading to fiercer competition between cacao and shade trees for light rather than for water and nutrients. A detailed study of root distribution patterns in Cacao-*Gliricidia* agroforests in nearby plantations have shown that the root systems of cacao and *Gliricidia* are vertically stratified with cacao roots concentrating in the upper profile and *Gliricidia* roots in the subsoil [44]. Indeed, stable isotope analyses confirmed that these two species showed complementary soil water use in these plantations [20]. Another study from Lehmann et al. [45] revealed that shaded crops like coffee and cacao tend to have shallower root activity than fruit trees and that most of the root activity of cacao trees occurs in the topsoil. A consequence is high complementary in the use of soil water reserves and thus, only limited competition between cacao and the shade trees [20,44,46,47]. However, for the more complex rooting patterns in our Cacao-multi systems, corresponding information on complementary root distribution and water partitioning is not yet available.

Likewise to tree biomass and the corresponding C stocks, carbon sequestration rates were significantly highest in Cacao-multi plots with 18 Mg C ha^-1^ yr^-1^ compared to the less productive Cacao-*Gliricidia* and Cacao-mono stands with 13 Mg C ha^-1^ yr^-1^ and 9 Mg C ha^-1^ yr^-1^, respectively. The productivity data for cacao trees show that increased shading resulted in preferential allocation of carbon toward aboveground stem growth and not to root growth: Fine root production of cacao tended to decrease (from 0.7 to 0.5 Mg C ha^-1^ yr^-1^) from the monocultures to the multi-shade tree systems, while coarse root growth slightly rose and stem and branch wood production also tended to increase (from 1.1 to 1.3 Mg C ha^-1^ yr^-1^). Thus, it appears that increased crowding in the canopy and root zone due to higher cacao stem densities in the shaded systems does not increase the belowground productivity of the individual tree. This holds support that the presence of shade trees may not lead to pronounced increasing light competition in the stand as well as it appears that the presence of shade trees does not induce increased belowground competition since fine root production is expected to increase under enhanced belowground competition due to increasing fine root turnover [48]. As *Theobroma cacao* is a C_3_-plant that is adapted to semi-shade in the forest understory, full sunlight may represent a stress factor of growth rather than a stimulating factor. Photosynthesis has been found to saturate in this species already at photon flux densities of ca. 400 μmol m^-2^ s^-1^ [49,50], which is equivalent to about 25% of full sunlight. Our data on aboveground woody biomass production in systems differing in shade intensity fit to the results of other studies that found stable or even increased vegetative growth of cacao trees cultivated under shade tree cover [24,43,51]. Köhler et al. [52] found enhanced water uptake of cacao and associated with it higher stem diameters and leaf areas in plantations shaded by *Gliricidia* than in monocultures in our study region. In a recent study from Köhler et al. [23], who investigated sap flux in the same study sites, a trend for higher water use of cacao trees grown under shade was reported as well.

Our data further show that the total NPP of cacao on the plot level decreased with increasing shade intensity (from 9.1 to 7.4 Mg C ha^-1^ yr^-1^) due to decreasing litter and root production, whereas cacao bean yield did not decrease. The stable bean production of approx. 2 Mg d. m. ha^-1^ yr^-1^ (1.2 Mg C ha^-1^ yr^-1^) across the three cultivation systems is relatively high compared to other studies (e.g. [53,54]), but match results of Ruf et al. [55], who reported a yield of marketable cacao beans of 2 Mg ha^-1^ for Sulawesi. In the literature, mixed results exist with respect to cacao bean yield change under increased shade tree cover. While a number of studies found a decrease [13,14,18,21,56–58], others reported no negative shade tree effect on yield [54,59–62]. Clearly, the shade trees' species identity, the intensity of shading and the planting density of cacao are all influencing the result of agroforestry system comparisons. Nevertheless, our data seem to indicate that shading does not impede cacao productivity and yield in a significant way.

On the single-tree level, total NPP of cacao decreased with increasing shade cover from 22 to 13 kg tree^-1^, while cacao bean yield per tree tended to decrease from 2.4 to 1.6 kg tree^-1^ (both trends not significant at P < 0.05). Accordingly, aboveground NPP per cacao tree decreased by 42% from the Cacao-mono to the Cacao-multi system, while bean production decreased by 33%. This shows that the stable yield at the plot level was to a large part the consequence of the higher cacao stem densities in the shaded systems. However, since the trees’ yield loss was smaller than their NPP reduction, shading seems to have triggered a welcome allocation shift toward seed production, which is of economic interest.

### Beneficial Effects of Shade Trees

Shade trees may help to reduce the stress exposure of cacao to high evaporative demand and high radiation intensity. Miyaji et al. [63] found that cacao leaves have a shorter leaf longevity and thus, are shed earlier, when growing in the upper canopy with higher exposition to sunlight. Full light exposure can also lead to stomatal closure resulting from leaf water status deterioration, which may reduce photosynthetic activity and growth [49]. Such sensitive responses might be related to the natural occurrence of cacao in the understory of closed forests. Our data on leaf litter production fit into this picture. We measured a nearly twofold higher leaf litter production (5.3 vs. 2.9 Mg ha^-1^ yr^-1^) in the non-shaded than in the shaded systems, indicating shorter leaf longevity presumably as a stress response to drought and high solar radiation. The fact that the monocultures achieved their wood production and total NPP with a much higher leaf production and turnover, must be interpreted as a hint on elevated stress at the foliage level. Thus, strong evidence exists that growing cacao in non-shaded monocultures places the species beyond the range of optimal growing conditions.

In fact, it seems that the removal of shade trees increases cacao bean yield, if at all, only in the short-term [12,14,17,18,56], while it increases physiological stress and may reduce the stability of the system. Several authors argued that cacao agroforests with shade trees may produce lower, but stable yields and thus, are more productive in the long-term [12,14,17,18,56]. Moreover, shade trees seem to increase the productive lifetime of cacao trees [54,64] through the reduction of physical stress. In intensified cacao cultivation systems, yield tends to decrease after 15–20 years [12]. In our cacao plantations, the trees had an age of already 20–25 years. Given the relatively high bean yield of 2 Mg ha^-1^ yr^-1^, this shows that cacao can remain productive for quite long time spans under a more or less dense canopy of shade trees.

There are also economic reasons for farmers to cultivate cacao under moderate shade cover. Due to pronounced fluctuation of the cocoa price on the world market, farmers should have an interest in a stable production, even at a perhaps somewhat reduced yield level, but at lower cost, compared to higher short-term yield with high input of labor and costs (e. g. [21]). Moreover, shaded and more complex-structured cacao agroforestry systems provide a number of important ecosystem services that may increase the farmer’s revenue and might be able to compensate possible negative effects (e.g. [12,13,21,23,65,66]).

A cover of shade trees, especially when it contains more than one tree species, harbors not only a more diverse fauna [19,67] than cacao monocultures, but it also provides additional non-monetary and monetary goods and services that need consideration, when selecting the most appropriate cultivation system. Nutrient input through nitrogen fixation by *Gliricidia* and other leguminous shade trees present on the shaded plots is likely an important N source in the Cacao-*Gliricidia* and the Cacao-multi systems. Nutrient cycling and nutrient addition to the topsoil is increased by a higher aboveground litter mass and accelerated decomposition due to N-rich litter. Pests and diseases cause huge losses in the cacao yield worldwide [68]. Diverse plant and animal communities may provide natural pest control through the provision of niches for insectivorous birds, parasitoids and pest-feeding insects (e.g. [13,14,57,69,70]). Wielgoss et al. [71] could show that cacao yield loss was reduced due to the co-existence of a minor pest, the mirid bug *Helopeltis sulawesi*, and a major pest, the pod-boring moth *Conopomorpha cramerella*, in a shaded cacao system. Although another major pest, the black pod disease, which is caused by *Phytophthora palmivora*, generally tends to increase with increasing humidity [21], it may be hold in check by the presence of more endophytic antagonists under a more diverse tree canopy [72,73]. Our measurements further show that relative air humidity was increased only very slightly with increasing shade tree cover, suggesting that *Phythopthora* should not profit significantly from the presence of shade trees in our stands and shade trees might act more like a protection from wind dispersal. A layer of shade trees can reduce weed cover under the cacao trees and may minimize soil erosion after heavy rainfall [12]. Shade trees and the associated entomofauna may also indirectly increase cacao bean yield by enhancing pollination services. This may be of particular relevance for the strictly entomophilous cacao, since pollinator abundance has been found to positively correlate with pod set and thus yield [74–76]. While the monetary value of most of these services is not exactly known, Obiri et al. [64] found highest net cash flow in shaded agroforests, where additional income was generated from the harvest of timber and other merchantable goods, and because labor and input costs were smaller than in intensively managed monocultures. Bisseleua et al. [57] showed for West African cacao plantations that the higher input needed for intensification not necessarily resulted in higher net returns to the farmer. In the light that cacao monocultures are suffering more from long-term yield reduction due to soil fertility loss, and are more susceptible to herbivore attack and disease infestations [12,17] and apparently also to drought than shaded cacao systems ([20], this study), farmers should be encouraged to choose cultivation systems with diverse shade trees instead of monocultures, where possible.

## Conclusion

The present study provides additional evidence that cacao bean yield does not necessarily decrease under a cover of shade trees and that shade seems to reduce physical stress. As demonstrated in our study, somewhat lower fruit production per tree under shade can be compensated by higher tree numbers and the provision of ecosystem services such as enhanced pollination success, biological pest and weed control, increased nitrogen input by legume shade trees and enhanced nutrient cycling with litter fall, as well as a reduced atmospheric vapor demand. Moreover, farmers profit from additional income provided by the harvest of timber, fruits and fuel wood provided by the shade trees. Shade trees could also lead to additional income, when charged within the REDD+ (Reducing Emissions from Deforestation and Forest Degradation) scheme or other certification programs. Our results demonstrate the carbon storage and sequestration potential, which is associated with a shade tree cover in cacao cultivation systems. The additional income and lower labor and input costs make cacao production stable to highly fluctuating cacao prices and more attractive by compensating farmers for possibly lower yields in the short-time compared to monocultures. If planned properly, shaded cacao plantations allow combining high yield with benefits for carbon sequestration, production system stability, and biodiversity

## Supporting Information

S1 Appendix Declaration to copyright of Fig 1. (PDF)

S1 Table Aboveground stand structural properties. Aboveground stand structural properties of the nine study sites of the three cultivation systems in the Kulawi valley (means per plot).
(PDF)

S2 Table Above- and belowground biomass stocks. Above- and belowground biomass stocks and the shoot:root ratio of the nine study sites of the three cultivation systems in the Kulawi valley (means per plot). Only for the group ‘all’ fine root data is included.
(PDF)

S3 Table Above- and belowground carbon stocks. Above- and belowground carbon stocks and the shoot:root carbon ratio of the nine study sites of the three cultivation systems in the Kulawi valley (means per plot). Only for the group ‘all’ fine root data is included.
(PDF)

S4 Table Net primary production (NPP). Components of annual net primary production (NPP) (in Mg ha^-1^ yr^-1^) of the nine study sites of the three cultivation systems in the Kulawi valley (means per plot). Note that coarse root biomass production includes production of root stocks as well.
(PDF)

S5 Table Carbon pools in net primary production (NPP). Associated carbon pools (in Mg C ha^-1^ yr^-1^) in annual net primary production (NPP) of the nine study sites of the three cultivation systems in the Kulawi valley (means per plot). Note that coarse root biomass production includes production of root stocks as well.
(PDF)
