# Supplementary material for: Cacao Cultivation under Diverse Shade Tree Cover Allows High Carbon Storage and Sequestration without Yield Losses
Source: PLoS One. 2016 Feb 29;11(2):e0149949. doi: 10.1371/journal.pone.0149949 (PMC4771168; doi:10.1371/journal.pone.0149949)
Supplement: S1 Appendix — (PDF) [file pone.0149949.s001.pdf]

Dear Editors,

I, Yasmin Abou Rajab, am the copy right holder. Figure 1 was created for the publication “Cacao cultivation under diverse shade tree cover allows high carbon storage and sequestration without yield losses “ and has not been published elsewhere. Figure 1 was created using QGIS Version 2.12.0.

Kind regards,

Yasmin Abou Rajab
