# Supplementary material for: Cacao Cultivation under Diverse Shade Tree Cover Allows High Carbon Storage and Sequestration without Yield Losses
Source: PLoS One. 2016 Feb 29;11(2):e0149949. doi: 10.1371/journal.pone.0149949 (PMC4771168; doi:10.1371/journal.pone.0149949)
Supplement: S1 Table — Aboveground stand structural properties of the nine study sites of the three cultivation systems in the Kulawi valley (means per plot). (PDF) [file pone.0149949.s002.pdf]

S1 Table. Aboveground stand structural properties. Aboveground stand structural properties of the nine study sites of the three cultivation systems in the Kulawi valley (Sulawesi, Indonesia) (means per plot).

**Cultivation system**	**Plot**	**Tree identity**	**Canopy cover** (%)	**Tree density** (no. ha^{-1})	**Stem density** (no. ha^{-1})	**Stand basal area** (m^2 ha^{-1})	**Stem diameter** (cm)	**Tree height** (m)	**Shannon-Index H'**
Cacao-mono	Plot 1	Cacao		640	1280	9.01	9.03	5.16	
Cacao-mono	Plot 2	Cacao		1055	2012	12.06	7.60	5.14	
Cacao-mono	Plot 3	Cacao		982	2118	16.85	9.22	5.11	
Cacao-mono	Plot 1	All	20	640	1280	9.01	9.03	5.16	0
Cacao-mono	Plot 2	All	60	1055	2012	12.06	7.60	5.14	0
Cacao-mono	Plot 3	All	70	982	2118	16.85	9.22	5.11	0
Cacao-*Gliricidia*	Plot 4	Cacao		992	2224	10.52	7.30	4.94	
Cacao-*Gliricidia*	Plot 5	Cacao		1330	3498	9.67	5.50	4.39	
Cacao-*Gliricidia*	Plot 6	Cacao		820	1892	7.48	6.72	4.51	
Cacao-*Gliricidia*	Plot 4	Shade trees		160	784	4.00	7.64	10.89	
Cacao-*Gliricidia*	Plot 5	Shade trees		494	644	5.66	6.64	7.56	
Cacao-*Gliricidia*	Plot 6	Shade trees		631	725	3.66	7.83	7.20	
Cacao-*Gliricidia*	Plot 4	All	40	1152	3008	14.52	7.39	5.76	0.17
Cacao-*Gliricidia*	Plot 5	All	80	1824	4142	15.33	6.14	5.25	0.25
Cacao-*Gliricidia*	Plot 6	All	60	1514	2681	11.56	7.08	5.77	0.36
Cacao-multi	Plot 7	Cacao		832	1520	11.33	8.53	5.08	
Cacao-multi	Plot 8	Cacao		1514	2940	15.40	7.29	5.35	
Cacao-multi	Plot 9	Cacao		1805	2644	15.37	8.00	4.83	
Cacao-multi	Plot 7	Shade trees		224	224	19.99	28.05	12.72	
Cacao-multi	Plot 8	Shade trees		592	1119	18.25	17.65	8.47	
Cacao-multi	Plot 9	Shade trees		254	280	23.54	28.16	14.47	
Cacao-multi	Plot 7	All	95	1056	1744	31.32	10.99	6.70	0.41
Cacao-multi	Plot 8	All	90	2107	4059	33.65	9.04	6.15	0.42
Cacao-multi	Plot 9	All	95	2060	2924	38.91	9.93	6.02	0.27
