# Supplementary material for: Cacao Cultivation under Diverse Shade Tree Cover Allows High Carbon Storage and Sequestration without Yield Losses
Source: PLoS One. 2016 Feb 29;11(2):e0149949. doi: 10.1371/journal.pone.0149949 (PMC4771168; doi:10.1371/journal.pone.0149949)
Supplement: S2 Table — Above- and belowground biomass stocks and the shoot:root ratio of the nine study sites of the three cultivation systems in the Kulawi valley (means per plot). Only for the group ‘all’ fine root data is included. (PDF) [file pone.0149949.s003.pdf]

S2 Table. Above- and belowground biomass stocks. Above- and belowground biomass stocks and the shoot:root ratio of the nine study sites of the three cultivation systems in the Kulawi valley (Sulawesi, Indonesia) (means per plot). Only for the group 'all' fine root data is included.

**Cultivation system**	**Plot**	**Tree identity**	**Aboveground biomass** (Mg ha^{-1})	**Coarse root biomass** (Mg ha^{-1})	**Fine root biomass** (Mg ha^{-1})	**Belowground biomass (note: just 'all' incl. fine roots)** (Mg ha^{-1})	**Total above- and belowground biomass** (Mg ha^{-1})	**ratio shoot : root (ratio AGB/BGB)**
Cacao-mono	Plot 1	Cacao	11.93	3.13		3.13	15.07	3.81
Cacao-mono	Plot 2	Cacao	15.97	4.16		4.16	20.13	3.84
Cacao-mono	Plot 3	Cacao	22.33	5.70		5.70	28.03	3.92
Cacao-mono	Plot 1	All	11.93	3.13	1.04	4.17	16.10	2.86
Cacao-mono	Plot 2	All	15.97	4.16	3.22	7.38	23.35	2.16
Cacao-mono	Plot 3	All	22.33	5.70	1.94	7.64	29.97	2.92
Cacao-*Gliricidia*	Plot 4	Cacao	13.91	3.78		3.78	17.69	3.67
Cacao-*Gliricidia*	Plot 5	Cacao	12.73	3.69		3.69	16.42	3.45
Cacao-*Gliricidia*	Plot 6	Cacao	9.87	2.79		2.79	12.66	3.54
Cacao-*Gliricidia*	Plot 4	Shade trees	19.00	4.47		4.47	23.47	4.25
Cacao-*Gliricidia*	Plot 5	Shade trees	19.34	4.31		4.31	23.66	4.48
Cacao-*Gliricidia*	Plot 6	Shade trees	13.74	3.37		3.37	17.11	4.08
Cacao-*Gliricidia*	Plot 4	All	32.91	8.25	3.13	11.38	44.29	2.89
Cacao-*Gliricidia*	Plot 5	All	32.07	8.00	3.57	11.57	43.64	2.77
Cacao-*Gliricidia*	Plot 6	All	23.61	6.16	2.36	8.52	32.13	2.77
Cacao-multi	Plot 7	Cacao	15.01	3.83		3.83	18.84	3.92
Cacao-multi	Plot 8	Cacao	20.37	5.41		5.41	25.78	3.77
Cacao-multi	Plot 9	Cacao	20.34	5.41		5.41	25.75	3.76
Cacao-multi	Plot 7	Shade trees	100.20	15.45		15.45	115.65	6.48
Cacao-multi	Plot 8	Shade trees	61.10	11.20		11.20	72.30	5.46
Cacao-multi	Plot 9	Shade trees	85.89	14.56		14.56	100.45	5.90
Cacao-multi	Plot 7	All	115.20	19.28	6.48	25.76	140.97	4.47
Cacao-multi	Plot 8	All	81.47	16.61	3.01	19.62	101.09	4.15
Cacao-multi	Plot 9	All	106.23	19.97	3.45	23.42	129.65	4.54
