# Supplementary material for: Cacao Cultivation under Diverse Shade Tree Cover Allows High Carbon Storage and Sequestration without Yield Losses
Source: PLoS One. 2016 Feb 29;11(2):e0149949. doi: 10.1371/journal.pone.0149949 (PMC4771168; doi:10.1371/journal.pone.0149949)
Supplement: S3 Table — Above- and belowground carbon stocks and the shoot:root carbon ratio of the nine study sites of the three cultivation systems in the Kulawi valley (means per plot). Only for the group ‘all’ fine root data is included. (PDF) [file pone.0149949.s004.pdf]

S3 Table. Above- and belowground carbon stocks. Above- and belowground carbon stocks and the shoot : root carbon ratio of the nine study sites of the three cultivation systems in the Kulawi valley (Sulawesi, Indonesia) (means per plot). Only for the group 'all' fine root data is included.

**Cultivation system**	**Plot**	**Tree identity**	**Aboveground carbon stock** (Mg ha^{-1})	**Coarse root carbon stock** (Mg ha^{-1})	**Fine root carbon stock** (Mg ha^{-1})	**Total belowground carbon stock (note: just 'all' incl. fine roots)** (Mg ha^{-1})	**Total above- and belowground carbon stock** (Mg ha^{-1})	**ratio shoot C : root C**
Cacao-mono	Plot 1	Cacao	5.52	1.32		1.32	6.83	4.19
Cacao-mono	Plot 2	Cacao	7.39	1.82		1.82	9.21	4.05
Cacao-mono	Plot 3	Cacao	10.33	2.60		2.60	12.93	3.97
Cacao-mono	Plot 1	All	5.52	1.32	0.45	1.77	7.28	3.12
Cacao-mono	Plot 2	All	7.38	1.82	1.43	3.26	10.64	2.27
Cacao-mono	Plot 3	All	10.33	2.60	0.85	3.45	13.78	2.99
Cacao-*Gliricidia*	Plot 4	Cacao	6.43	1.68		1.68	8.11	3.83
Cacao-*Gliricidia*	Plot 5	Cacao	5.89	1.54		1.54	7.43	3.82
Cacao-*Gliricidia*	Plot 6	Cacao	4.57	1.31		1.31	5.87	3.49
Cacao-*Gliricidia*	Plot 4	Shade trees	9.18	1.62		1.62	10.80	5.66
Cacao-*Gliricidia*	Plot 5	Shade trees	9.34	2.35		2.35	11.69	3.97
Cacao-*Gliricidia*	Plot 6	Shade trees	6.64	2.31		2.31	8.95	2.87
Cacao-*Gliricidia*	Plot 4	All	15.61	3.50	1.34	4.84	20.45	3.23
Cacao-*Gliricidia*	Plot 5	All	15.23	3.25	1.44	4.69	19.91	3.25
Cacao-*Gliricidia*	Plot 6	All	11.20	2.71	0.98	3.69	14.89	3.03
Cacao-multi	Plot 7	Cacao	6.94	1.82		1.82	8.76	3.81
Cacao-multi	Plot 8	Cacao	9.42	1.71		1.71	11.13	5.52
Cacao-multi	Plot 9	Cacao	9.41	1.41		1.41	10.81	6.69
Cacao-multi	Plot 7	Shade trees	47.43	6.45		6.45	53.88	7.35
Cacao-multi	Plot 8	Shade trees	28.93	4.84		4.84	33.76	5.98
Cacao-multi	Plot 9	Shade trees	40.66	6.33		6.33	46.99	6.42
Cacao-multi	Plot 7	All	54.37	8.07	2.77	10.85	65.22	5.01
Cacao-multi	Plot 8	All	38.35	7.19	1.16	8.35	46.70	4.59
Cacao-multi	Plot 9	All	50.06	8.64	1.44	10.08	60.14	4.97
