# Supplementary material for: Cacao Cultivation under Diverse Shade Tree Cover Allows High Carbon Storage and Sequestration without Yield Losses
Source: PLoS One. 2016 Feb 29;11(2):e0149949. doi: 10.1371/journal.pone.0149949 (PMC4771168; doi:10.1371/journal.pone.0149949)
Supplement: S5 Table — Associated carbon pools (in Mg C ha-1 yr-1) in annual net primary production (NPP) of the nine study sites of the three cultivation systems in the Kulawi valley (means per plot). Note that coarse root biomass production includes production of root stocks as well. (PDF) [file pone.0149949.s006.pdf]

S5 Table. Carbon pools in net primary production (NPP). Associated carbon pools (in Mg C ha^{-1} yr^{-1}) in annual net primary production (NPP) of the nine study sites of the three cultivation systems in the Kulawi valley (Sulawesi, Indonesia) (means per plot). Note that coarse root biomass production includes production of root stocks as well.

**Cultivation system**	**Plot**	**Tree identity**	**C in Cacao bean yield**	**C in Cacao fruit production**	**C in Above-ground woody biomass production**	**C in Litter production**	**C in Fine root production** (0-60cm)	**C in Coarse root biomass production**	**C in Total aboveground production**	**C in Total belowground production**	**C in Total production**
Cacao-mono	Plot 1	Cacao	0.88	2.29	0.81	1.86	0.23	0.17	5.35	4.95	0.40
Cacao-mono	Plot 2	Cacao	1.22	5.30	0.65	2.40	1.52	0.14	10.02	8.36	1.66
Cacao-mono	Plot 3	Cacao	1.46	6.36	1.78	3.00	0.48	0.40	12.01	11.13	0.88
Cacao-mono	Plot 1	All			0.81	1.86	0.23	0.17	5.35	4.95	0.40
Cacao-mono	Plot 2	All			0.65	2.40	1.52	0.14	10.02	8.36	1.66
Cacao-mono	Plot 3	All			1.78	3.00	0.48	0.40	12.01	11.13	0.88
Cacao-*Gliricidia*	Plot 4	Cacao	1.09	4.89	1.88	1.08	0.61	0.43	8.88	7.84	1.04
Cacao-*Gliricidia*	Plot 5	Cacao	1.79	7.94	1.15	1.84	0.53	0.26	11.72	10.93	0.79
Cacao-*Gliricidia*	Plot 6	Cacao	0.69	3.49	1.28	1.11	0.62	0.32	6.82	5.89	0.93
Cacao-*Gliricidia*	Plot 4	Shade trees			3.74	0.71	0.46	0.64	5.56	4.45	1.11
Cacao-*Gliricidia*	Plot 5	Shade trees			2.83	1.18	0.19	0.45	4.65	4.01	0.64
Cacao-*Gliricidia*	Plot 6	Shade trees			1.72	0.60	0.06	0.31	2.69	2.32	0.37
Cacao-*Gliricidia*	Plot 4	All			5.62	1.79	1.07	1.07	14.44	10.83	1.83
Cacao-*Gliricidia*	Plot 5	All			3.98	3.02	0.72	0.71	16.37	14.79	2.04
Cacao-*Gliricidia*	Plot 6	All			3.00	1.72	0.67	0.63	9.51	9.82	1.01
Cacao-multi	Plot 7	Cacao	1.27	5.20	0.56	1.14	0.59	0.12	7.60	6.90	0.70
Cacao-multi	Plot 8	Cacao	0.43	1.93	2.20	1.23	0.25	0.48	6.09	5.36	0.73
Cacao-multi	Plot 9	Cacao	1.67	5.09	1.12	1.55	0.59	0.24	8.59	7.76	0.83
Cacao-multi	Plot 7	Shade trees			4.81	3.44	0.15	0.59	9.00	8.26	0.74
Cacao-multi	Plot 8	Shade trees			6.91	3.70	0.22	0.99	11.83	10.61	1.21
Cacao-multi	Plot 9	Shade trees			6.41	2.56	0.09	0.92	9.97	8.97	1.01
Cacao-multi	Plot 7	All			5.37	4.58	0.74	0.70	16.60	10.32	1.44
Cacao-multi	Plot 8	All			9.12	4.92	0.47	1.48	17.92	12.86	1.57
Cacao-multi	Plot 9	All			7.53	4.11	0.68	1.16	18.56	24.67	2.22
